# Social presence during brief immersive virtual concert viewing: an experimental comparison of audience enjoyment, emotional arousal, and post-exposure fan identification

**DOI:** 10.3389/fpsyg.2026.1928328

**Published:** 2026-10-13

**Authors:** Xiaobin Li, Meiji Huo

**Affiliations:** Beihua University, Jilin, China

**Keywords:** emergent fan identification, emotional arousal, enjoyment, immersive virtual concert, media psychology, social presence, virtual reality

## Abstract

Immersive virtual concert viewing is becoming an increasingly important form of digital music experience, yet the psychological processes associated with audience responses in such environments remain insufficiently understood. This study examined whether social presence statistically accounted for differences in audience responses between two concert-viewing configurations. A laboratory experiment was conducted with 240 participants aged 18–30 years, who were randomly assigned to either an immersive virtual concert condition or a standard two-dimensional concert video condition. After viewing a 4-min concert clip, participants completed measures of perceived immersiveness, social presence, enjoyment, emotional arousal, and emergent fan identification. The immersive virtual concert condition produced significantly higher perceived immersiveness and social presence than the standard video condition. Social presence was positively associated with enjoyment, emotional arousal, and emergent fan identification. Bootstrap indirect-effect analyses further showed significant indirect associations between viewing condition and all three audience outcomes through social presence. These findings provide preliminary evidence that social presence is associated with the enhanced audience responses observed under a brief immersive concert-viewing configuration. Because the experimental conditions bundled several technological differences and social presence was not independently manipulated, the findings should not be interpreted as isolating a specific technological feature or establishing a causal mediating mechanism.

## 1 Introduction

Digital media technologies have increasingly transformed the ways in which audiences encounter, consume, and participate in music. These formats do not simply relocate concerts from physical venues to digital screens; rather, they reshape the psychological conditions under which audiences experience musical performance. In a traditional concert, enjoyment is partly supported by spatial co-presence, collective audience response, bodily engagement, and the perceived immediacy of the performer. In the immersive concert-viewing condition, these elements are technologically mediated, raising an important media-psychological question: under what conditions can a mediated concert experience become psychologically vivid, socially meaningful, and emotionally engaging?

Recent studies suggest that virtual and livestreamed concerts can generate distinctive audience experiences, but the psychological processes associated with these audience experiences remain insufficiently understood. Onderdijk et al. (2023) reported that virtual reality concert attendees often valued the uniqueness and accessibility of VR concerts, and that positive evaluations of VR concert experiences were influenced by experienced immersivity. Similarly, Lo and Lai (2023) experimentally demonstrated that immersive VR and active navigation could enhance presence, flow, satisfaction, and concert-attending intention in virtual concert contexts. Kulla et al. (2024) further showed that livestreamed concert viewing produced higher levels of social presence and enjoyment than recorded concert viewing, although the mediating role of social presence was not fully supported. Together, these findings indicate that virtual concert formats can improve audience experience, but they also suggest that further experimental work is needed to clarify how technological immersion is translated into psychological outcomes.

A useful concept for addressing this question is social presence. Social presence refers to the subjective sense of being together with, connected to, or socially aware of others in a mediated environment. In the context of music, this construct is especially important because musical experience is often inherently social. Van Kerrebroeck et al. (2021) argued that VR offers new possibilities for studying social music cognition because it can create the illusion of being and interacting with others inside a virtual environment. From this perspective, the psychological value of the immersive concert-viewing condition may not depend solely on audiovisual realism, but on whether the medium allows viewers to feel socially present with performers and co-audience members.

Social presence may be particularly relevant for explaining three central outcomes of virtual concert experience: enjoyment, emotional arousal, and emergent fan identification. First, enjoyment is a core indicator of media entertainment and audience satisfaction. A virtual concert that feels socially alive may be more enjoyable because viewers perceive the event as a shared performance rather than a solitary video-watching experience. Second, emotional arousal reflects the intensity of the audience's affective response. Music already has strong emotion-eliciting potential, but a heightened sense of being “there” with performers and others may amplify excitement, stimulation, and emotional involvement. Third, emergent fan identification captures the extent to which viewers feel connected to the performer, the fan community, or the symbolic identity associated with the musical event. This outcome is important because contemporary music consumption increasingly occurs within digital fan cultures, where identity, belonging, and mediated interaction are closely intertwined.

The inclusion of emergent fan identification also extends virtual concert research beyond immediate enjoyment. In digital media environments, fans do not only consume performances; they interpret, share, comment on, and use them to express identity and group belonging. Recent research on K-pop fandom suggests that fandom identity is associated with social connectedness and subjective wellbeing (Arizabal and Yabut, 2025). In the context of virtual idols, Liu (2025) found that parasocial relationships contributed to fan community participation, indicating that mediated performers can become meaningful objects of social and emotional attachment. These findings imply that immersive concert media may influence not only how much audiences enjoy a performance, but also how strongly they identify with performers and fan communities. However, relatively little experimental research has examined whether social presence statistically accounts for differences in enjoyment, emotional arousal, and post-exposure fan identification across immersive and non-immersive concert-viewing configurations.

The present study addresses this gap by experimentally examining the role of social presence in immersive virtual concert experience. Specifically, participants are assigned to watch either an immersive virtual concert clip or a standard two-dimensional concert video. After viewing the stimulus, they report their perceived social presence, enjoyment, emotional arousal, and emergent fan identification. This design allows us to test whether an immersive virtual concert format produces stronger social presence than a conventional video format and whether social presence predicts key audience outcomes.

Based on prior research on VR concerts, livestreamed concerts, social presence, and digital fan engagement, we proposed three hypotheses. First, participants in the immersive virtual concert condition were expected to report higher social presence than those in the standard concert video condition. Second, social presence was expected to be positively associated with enjoyment, emotional arousal, and emergent fan identification. Third, we expected statistically significant indirect associations between concert-viewing condition and these audience outcomes through social presence. Because social presence was measured rather than experimentally manipulated, this third hypothesis concerns statistical indirect effects rather than a causal mediating mechanism.

For the present study, emergent fan identification was operationalized as an immediate, state-like, post-exposure identification response rather than as an established or enduring fan identity. Accordingly, higher scores reflect stronger short-term feelings of connection with the performer and a music-related fan community and should not be interpreted as evidence that a new and stable fan identity was formed during the brief viewing episode.

## 2 Method

### 2.1 Participants

Participants were recruited through university advertisements and campus social media. Eligible participants were required to be between 18 and 30 years old, have normal or corrected-to-normal vision and hearing, and have prior experience with online music listening or concert video viewing. Participants who reported discomfort during VR viewing, technical difficulties, failed attention-check items, or completed the questionnaire in an unrealistically short time were excluded.

A total of 268 participants initially entered the study and were randomly assigned to one of the two viewing conditions. Following data-quality screening, 28 participants were excluded because they failed attention checks, reported substantial discomfort during VR viewing, experienced technical problems that disrupted stimulus presentation, or completed the study in less than one-third of the median completion time. Of these excluded participants, 14 had been assigned to the immersive virtual concert condition and 14 to the standard two-dimensional video condition. The final analytic sample therefore consisted of 240 participants, including 142 women and 98 men, with a mean age of 21.46 years (SD = 2.37). The final sample included 120 participants in the immersive virtual concert condition and 120 in the standard two-dimensional video condition.

### 2.2 Design and experimental stimuli

The study used a single-factor between-subjects design with two viewing conditions: an immersive virtual concert condition and a standard two-dimensional concert video condition. The experimental stimulus consisted of a 4-min excerpt from a 360-degree recording of the same musical performance. The source material was recorded during an actual live concert rather than staged specifically for the experiment or digitally composited. A live audience was present during the performance, and other audience members were visible within portions of the 360-degree visual field. The selected excerpt contained a continuous segment of one performance and was identical across conditions with respect to performer, song, performance take, temporal segment, duration, and audio content.

Both experimental versions were derived from the same 360-degree master recording. In the immersive virtual concert condition, participants viewed the original 360-degree version through a head-mounted display from an audience-centered perspective. The full surrounding visual field was retained, and participants could change their viewing direction naturally through head movements during playback. In the standard two-dimensional condition, a fixed front-facing field of view was extracted from the same 360-degree master recording and presented as a conventional two-dimensional video on a computer monitor. The standard version therefore preserved the same musical performance and temporal sequence while removing the panoramic visual field and head-contingent viewpoint exploration.

The use of a common master recording was intended to minimize differences in musical and performance content between conditions. Both versions had the same 4-min duration and identical audio track and presented the same performer, song, and performance segment. Thus, differences between the two conditions did not reflect different performances or different musical material.

At the same time, the experimental manipulation should be understood as a comparison between two complete viewing configurations rather than as an isolated manipulation of a single technological feature. The immersive condition combined presentation through a head-mounted display, 360-degree visual presentation, a broader available visual field, and head-contingent viewpoint exploration, whereas the standard condition combined presentation on a computer monitor, a fixed two-dimensional frame, and no viewpoint control. Consequently, the present design cannot independently determine whether any observed between-condition difference is attributable specifically to the display device, panoramic video format, field of view, viewpoint control, or some combination of these features.

A pilot test was conducted with 30 participants who were not included in the main experiment. The pilot test confirmed that the immersive version was perceived as significantly more immersive than the standard two-dimensional version, whereas the two versions did not differ significantly in perceived music quality, performer attractiveness, familiarity with the performer, familiarity with the song, familiarity with the musical genre, or liking of the musical style. These results supported the use of the two versions for comparing audience responses to the overall immersive and standard viewing configurations.

### 2.3 Procedure

Before stimulus exposure, participants completed demographic and background measures, including age, gender, prior virtual concert experience, frequency of music-streaming use, familiarity with the musical genre, familiarity with the performer, familiarity with the song, pre-existing fan status, and previous exposure to the specific stimulus video. Participants who reported previous exposure to the stimulus were also asked to indicate the approximate number of previous viewings.

Participants were then randomly assigned to one of the two conditions. In the immersive condition, participants viewed the concert using a Meta Quest 2 head-mounted display. Participants remained seated but were free to turn their heads to explore the surrounding 360-degree visual field. In the standard condition, participants viewed the fixed two-dimensional version on a 24-inch computer monitor at an approximately 60-cm viewing distance. All participants used the same model of headphones, and the audio track and playback duration were identical across conditions. All participants wore headphones and were instructed to avoid unrelated activities during viewing. After the clip, they completed measures of perceived immersiveness, social presence, enjoyment, emotional arousal, and emergent fan identification. Finally, they answered attention-check questions and were debriefed.

### 2.4 Measures

Unless otherwise stated, all multi-item measures were rated on a 7-point Likert scale ranging from 1 = strongly disagree to 7 = strongly agree. Higher scores indicated higher levels of the corresponding construct. The measures were adapted from established instruments in presence research, media enjoyment research, affective assessment, and fan studies. Because the present study focused on virtual concert experience, item wording was modified to fit the concert-viewing context.

Pre-exposure characteristics. Prior virtual concert experience was assessed with a dichotomous item asking whether participants had previously attended or viewed a virtual concert (0 = no, 1 = yes). Frequency of music-streaming use was assessed on a 7-point scale ranging from 1 = rarely or never to 7 = several times per day. Familiarity with the musical genre, familiarity with the performer, and familiarity with the song were each assessed before stimulus exposure using 7-point scales ranging from 1 = not at all familiar to 7 = extremely familiar. Pre-existing fan status was assessed using a dichotomous item asking whether participants considered themselves a fan of the performer before the experiment (0 = no, 1 = yes). Previous exposure to the specific stimulus video was assessed using a yes/no item. Participants who indicated that they had previously viewed the video were additionally asked to report the approximate number of previous viewings.

Perceived immersiveness. Perceived immersiveness was measured as a manipulation check with three items adapted from spatial presence and presence measures, including the MEC Spatial Presence Questionnaire and the Spatial Presence Experience Scale (Hartmann et al., 2016; Vorderer et al., 2004; Wirth et al., 2007). These instruments have been widely used to assess the extent to which media users feel located within a mediated environment. Example items included “The concert environment felt vivid and immersive,” “I felt surrounded by the concert environment,” and “I felt as if I was inside the concert space.” The scale showed good internal consistency, Cronbach's α =0.88 and McDonald's ω =0.89.

Social presence. Social presence was assessed with three items adapted from the Networked Minds Measure of Social Presence and the Temple Presence Inventory (Biocca et al., 2001; Lombard et al., 2009). The items measured the extent to which participants felt socially connected with the performer and other audience members during the concert experience. Example items included “I felt as if the performer was present with me,” “I felt connected with other audience members during the concert,” and “The concert felt like a shared social experience.” The scale showed excellent internal consistency, Cronbach's α =0.91 and McDonald's ω =0.92.

Enjoyment. Enjoyment was measured with four items adapted from media entertainment and enjoyment research. Enjoyment has been regarded as a central psychological outcome of media entertainment experience (Vorderer et al., 2004). Participants indicated the extent to which the concert experience was enjoyable, pleasant, entertaining, and satisfying. Example items included “I enjoyed watching the concert,” “The concert was entertaining,” and “Watching the concert was a pleasant experience.” The scale showed excellent internal consistency, Cronbach's α =0.90 and McDonald's ω =0.91.

Emotional arousal. Emotional arousal was measured with four adjective-based items adapted from affective assessment research on arousal (Bradley and Lang, 1994). Participants rated how excited, stimulated, energized, and emotionally moved they felt after watching the concert clip. Higher scores indicated stronger affective activation. The scale showed good internal consistency, Cronbach's α =0.86 and McDonald's ω =0.87.

Emergent fan identification. Emergent fan identification was assessed with three items adapted from the Fanship Scale and the Popular Media Fan Identity Scale (Reysen and Branscombe, 2010; Vinney et al., 2019). In the present study, the construct was operationalized as an immediate, state-like, post-exposure identification response rather than as a stable or enduring fan identity. The measure was intended to capture participants' immediate sense of connection with the performer and a music-related fan community following the viewing episode. Example items included “This concert made me feel closer to the performer,” “I felt connected to people who enjoy this type of music,” and “The concert strengthened my sense of belonging to a music-related fan community.” Higher scores therefore indicate stronger fan-related identification immediately following the experimental exposure and should not be interpreted as demonstrating the formation of a new, stable fan identity. The scale showed good internal consistency, Cronbach's α =0.89 and McDonald's ω =0.90.

### 2.5 Data analysis

Data were analyzed using SPSS 29.0 and the PROCESS macro. First, descriptive statistics, internal consistency coefficients, and Pearson correlations were calculated for all main variables. Cronbach's alpha and McDonald's omega were used to evaluate the reliability of multi-item scales. Second, independent-samples *t tests* were conducted to examine whether the immersive virtual concert condition produced higher perceived immersiveness and social presence than the standard concert video condition. The perceived immersiveness analysis served as the manipulation check.

Third, regression analyses were conducted to examine whether social presence was positively associated with enjoyment, emotional arousal, and emergent fan identification. In each model, the outcome variable was regressed on social presence. Finally, statistical mediation analyses with 5,000 bootstrap samples were conducted to estimate the indirect associations between concert-viewing condition and the three audience outcomes through social presence. Although assignment to viewing condition was randomized, social presence itself was not experimentally manipulated, and social presence and the outcomes were assessed within the same post-exposure measurement session. The indirect effects are therefore interpreted as statistical indirect effects and not as definitive evidence of causal mediation. Concert format was dummy-coded, with 0 representing the standard concert video condition and 1 representing the immersive virtual concert condition. Indirect effects were considered significant when the 95% bootstrap confidence interval did not include zero. As a robustness analysis, the mediation models were re-estimated while adjusting for pre-exposure familiarity with the performer, familiarity with the song, pre-existing fan status, and previous exposure to the specific stimulus video. These variables were entered simultaneously as covariates in both the mediator and outcome models. Bootstrap confidence intervals for the adjusted indirect effects were again estimated using 5,000 resamples. These analyses were conducted to examine whether the primary pattern of indirect associations remained after accounting for measured pre-exposure characteristics that could plausibly influence audience responses.

## 3 Results

### 3.1 Preliminary analyses

Preliminary analyses were conducted to examine whether the two experimental groups differed in demographic and background variables. No significant differences were found between the immersive virtual concert condition and the standard two-dimensional video condition in age, *t* (238) = 0.74, *p* = 0.459, gender distribution, χ^2^(1) = 0.30, *p* = 0.583, prior virtual concert experience, χ^2^(1) = 0.42, *p* = 0.517, or familiarity with the music genre, *t* (238) = 0.81, *p* = 0.419, or frequency of music-streaming use, *t* (238) = 0.67, *p* = 0.503. The groups also did not differ significantly in familiarity with the performer, *t* (238) = 0.52, *p* = 0.604; familiarity with the song, *t* (238) = 0.91, *p* = 0.364; pre-existing fan status, χ^2^(1) = 0.18, *p* = 0.671; or previous exposure to the specific stimulus video, χ^2^(1) = 0.65, *p* = 0.420. These results indicated that random assignment produced comparable groups before stimulus exposure. Across the full sample, familiarity with the performer averaged M = 2.68 (SD = 1.41), and familiarity with the song averaged M = 3.12 (SD = 1.56). A total of 42 participants (17.5%) identified themselves as pre-existing fans of the performer, and 26 participants (10.8%) reported having previously viewed the specific stimulus video. Among participants with previous exposure, the reported number of prior viewings was M = 2.15 (SD = 1.29, range = 1–6).

Descriptive statistics, internal consistency coefficients, and correlations among the main variables are presented in Table 1. All multi-item measures showed acceptable to excellent internal consistency, with Cronbach's α values ranging from .086 to 0.91. Perceived immersiveness was positively correlated with social presence, enjoyment, emotional arousal, and emergent fan identification. Social presence was also positively associated with all three audience outcomes, suggesting that participants who felt more socially present during the concert experience tended to report greater enjoyment, stronger emotional arousal, and higher emergent fan identification.

**Table 1:** Descriptive statistics, reliabilities, and correlations among main variables.

Variable	M	SD	α	ω	1	2	3	4	5
1. Perceived immersiveness	4.76	1.21	0.88	0.89	—				
2. Social presence	4.40	1.18	0.91	0.92	0.62^***^	—			
3. Enjoyment	4.89	1.08	0.90	0.91	0.58^***^	0.64^***^	—		
4. Emotional arousal	4.71	1.10	0.86	0.87	0.53^***^	0.59^***^	0.61^***^	—	
5. Emergent fan identification	4.18	1.23	0.89	0.90	0.47^***^	0.56^***^	0.52^***^	0.49^***^	—

*N* = 240. ^***^p < 0.001.

### 3.2 Manipulation check and group differences

An independent-samples *t test* was conducted to examine whether the experimental manipulation successfully increased perceived immersiveness. As expected, participants in the immersive virtual concert condition reported significantly higher perceived immersiveness (M = 5.42, SD = 0.94) than those in the standard two-dimensional video condition (M = 4.10, SD = 1.12), *t* (238) = 9.90, *p* < 0.001, Cohen's *d* = 1.28. This result confirmed the effectiveness of the experimental manipulation.

The immersive virtual concert condition also produced significantly higher social presence than the standard two-dimensional video condition. Participants in the immersive virtual concert condition reported stronger social presence (M = 4.97, SD = 1.00) than those in the standard two-dimensional video condition (M = 3.83, SD = 1.05), *t* (238) = 8.62, *p* < 0.001, Cohen's *d* = 1.11. Thus, Hypothesis 1 was supported.

Additional group comparisons showed that participants in the immersive virtual concert condition reported higher enjoyment, emotional arousal, and emergent fan identification than those in the standard two-dimensional video condition. Specifically, the immersive virtual concert condition produced greater enjoyment (M = 5.23, SD = 0.98) than the standard two-dimensional video condition (M = 4.54, SD = 1.05), *t*( 238) = 5.25, *p* < 0.001, Cohen's *d* = 0.68. Participants in the immersive virtual concert condition also reported stronger emotional arousal (M = 5.05, SD = 1.02) than those in the standard two-dimensional video condition (M = 4.36, SD = 1.06), *t* (238) = 5.14, *p* < 0.001, Cohen's *d* = 0.66. Finally, emergent fan identification was higher in the immersive virtual concert condition (M = 4.59, SD = 1.13) than in the standard two-dimensional video condition (M = 3.78, SD = 1.18), *t* (238) = 5.43, *p* < 0.001, Cohen's *d* = 0.70. These group differences are summarized in Table 2.

**Table 2:** Group differences between experimental conditions.

Variable	Standard two-dimensional video M(SD)	Immersive virtual concert M(SD)	*t*	*p*	Cohen's d
Perceived immersiveness	4.10 (1.12)	5.42 (0.94)	9.90	< 0.001	1.28
Social presence	3.83 (1.05)	4.97 (1.00)	8.62	< 0.001	1.11
Enjoyment	4.54 (1.05)	5.23 (0.98)	5.25	< 0.001	0.68
Emotional arousal	4.36 (1.06)	5.05 (1.02)	5.14	< 0.001	0.66
Emergent fan identification	3.78 (1.18)	4.59 (1.13)	5.43	< 0.001	0.70

### 3.3 Associations of social presence

Regression analyses were conducted to examine the associations between social presence and the three audience outcomes. Social presence was significantly and positively associated with enjoyment, β = 0.64, *t* (238) = 12.86, *p* < 0.001, emotional arousal, β = 0.59, *t* (238) = 11.24, *p* < 0.001, and emergent fan identification, β = 0.56, *t* (238) = 10.42, *p* < 0.001. These results indicated that participants who experienced stronger social presence during the concert reported greater enjoyment, stronger emotional arousal, and higher emergent fan identification. Therefore, Hypothesis 2 was supported.

### 3.4 Mediation analyses

Statistical indirect-effect analyses were conducted using 5,000 bootstrap samples to estimate whether differences between the two concert-viewing conditions in enjoyment, emotional arousal, and emergent fan identification were statistically accounted for through social presence. Concert format was dummy-coded, with 0 representing the standard two-dimensional video condition and 1 representing the immersive virtual concert condition.

For enjoyment, concert format significantly predicted social presence, B = 1.14, SE = 0.13, *p* < 0.001, and social presence was significantly associated with enjoyment after controlling for concert format, B = 0.55, SE = 0.05, *p* < 0.001. The indirect effect of concert format on enjoyment through social presence was significant, B = 0.63, SE = 0.10, 95% CI [0.44, 0.84]. The direct effect of concert format on enjoyment became non-significant after social presence was included in the model, B = 0.06, SE = 0.12, *p* = 0.610, indicating a significant indirect effect with a non-significant direct effect.

For emotional arousal, social presence was significantly associated with emotional arousal after controlling for concert format, B = 0.50, SE = 0.06, *p* < 0.001. The indirect effect of concert format on emotional arousal through social presence was significant, B = 0.57, SE = 0.11, 95% CI [0.37, 0.81]. The direct effect of concert format on emotional arousal was not significant after social presence was included, B = 0.12, SE = 0.13, *p* = 0.358, indicating a significant indirect effect with a non-significant direct effect.

For emergent fan identification, social presence was significantly associated with emergent fan identification after controlling for concert format, B = 0.48, SE = 0.07, *p* < 0.001. The indirect effect of concert format on emergent fan identification through social presence was significant, B = 0.55, SE = 0.12, 95% CI [0.34, 0.78]. The direct effect of concert format on emergent fan identification remained significant, B = 0.26, SE = 0.13, *p* = 0.048, indicating a significant indirect effect with a significant direct effect. These findings supported the predicted indirect associations. However, because social presence was measured rather than experimentally manipulated, the results should be interpreted as evidence of statistically significant indirect effects rather than as establishing a causal mediating mechanism. The bootstrap indirect effects are summarized in Table 3.

**Table 3:** Bootstrap indirect effects of concert-viewing condition on audience outcomes through social presence.

Outcome	Indirect effect B	SE	95% CI
Enjoyment	0.63	0.10	[0.44, 0.84]
Emotional arousal	0.57	0.11	[0.37, 0.81]
Emergent fan identification	0.55	0.12	[0.34, 0.78]

Concert format was coded as 0 = standard two-dimensional video and 1 = immersive virtual concert. Bootstrap samples = 5,000. Confidence intervals that do not include zero indicate significant indirect effects.

### 3.5 Robustness analyses

To examine whether the indirect associations were robust to participants' pre-exposure characteristics, the mediation models were re-estimated while adjusting simultaneously for familiarity with the performer, familiarity with the song, pre-existing fan status, and previous exposure to the specific stimulus video. The substantive pattern of results remained unchanged. Viewing condition remained significantly associated with social presence after adjustment, B = 1.10, SE = 0.14, *p* < 0.001. Social presence remained significantly associated with enjoyment, B = 0.53, SE = 0.05, *p* < 0.001; emotional arousal, B = 0.48, SE = 0.06, *p* < 0.001; and emergent fan identification, B = 0.46, SE = 0.07, *p* < 0.001.

The adjusted indirect effect through social presence remained statistically significant for enjoyment, B = 0.58, SE = 0.10, 95% CI [0.40, 0.79]; emotional arousal, B = 0.53, SE = 0.10, 95% CI [0.34, 0.74]; and emergent fan identification, B = 0.51, SE = 0.11, 95% CI [0.30, 0.72]. Thus, the indirect-effect pattern remained evident after accounting for participants' prior familiarity, existing fan status, and previous exposure to the stimulus.

## 4 Discussion

The present study examined whether social presence statistically accounted for part of the association between concert format and audience experience. The findings supported the proposed hypotheses. Compared with participants who watched a standard two-dimensional video, those in the immersive virtual concert condition reported higher perceived immersiveness and stronger social presence. Social presence was positively associated with enjoyment, emotional arousal, and emergent fan identification, and mediation analyses showed significant indirect effects of concert format on all three outcomes through social presence. These findings indicate that the immersive viewing configuration was associated with higher perceived immersiveness, stronger social presence, and more positive audience responses than the standard two-dimensional viewing configuration. Social presence statistically accounted for part of these between-condition differences. Because the experimental conditions simultaneously differed in display device, video format, field of view, and viewpoint control, however, the present study cannot determine which individual technological feature produced these differences. Likewise, the indirect-effects analyses do not establish social presence as a causal mediator.

The finding that the immersive concert-viewing condition produced higher social presence is consistent with previous research showing that VR-based and immersive concert formats can increase presence, flow, satisfaction, and concert-related intentions (Lo and Lai, 2023; Onderdijk et al., 2021, 2023). It also aligns with studies of livestreamed and virtual concerts suggesting that mediated concert formats can influence social connection, enjoyment, and emotional responses (Kulla et al., 2024; Swarbrick et al., 2021). Concerts are inherently social events: audiences do not only listen to music, but also attend to performers, perceive collective responses, and experience a sense of shared participation. The present results indicate that the immersive concert-viewing condition may partially reconstruct this social atmosphere in mediated form.

Social presence was also positively associated with enjoyment and emotional arousal. This finding supports media entertainment theory, which regards enjoyment as a central psychological outcome of media use (Vorderer et al., 2004), and presence research suggesting that immersive media can intensify users' involvement in mediated environments (Sterna and Zibrek, 2021; Wirth et al., 2007). In the context of virtual concerts, social presence may make the experience feel less like solitary video watching and more like participation in a shared musical event. This sense of being socially connected may increase both the pleasantness of the experience and the intensity of emotional activation.

Another finding was that social presence was positively associated with emergent fan identification. Importantly, this outcome should be interpreted as an immediate, state-like post-exposure identification response rather than as evidence that a stable fan identity was formed during the 4-min viewing period. Participants who experienced stronger social presence reported a greater immediate sense of connection with the performer and the broader music-related fan community. Whether such short-term responses persist, accumulate across repeated exposures, or develop into relatively stable fan identification cannot be determined from the present design. Previous research has shown that fandom is associated with belonging, emotional attachment, and social connectedness (Reysen and Branscombe, 2010; Vinney et al., 2019). Recent studies on digital fandom and virtual idols further suggest that mediated performers and online fan communities can support parasocial relationships and community participation (Arizabal and Yabut, 2025; Kim et al., 2023; Liu, 2025). The present findings indicate that higher post-exposure fan-identification responses in the immersive viewing condition were statistically associated with stronger social presence. Whether social presence contributes causally to the development of these short-term identification responses requires further experimental investigation.

Theoretically, this study contributes to media psychology by identifying social presence as a potential psychological process associated with differences in audience responses across concert-viewing configurations. Prior studies have shown that virtual concerts can be engaging and emotionally meaningful, but less work has directly tested why immersive concert formats produce these effects. By showing that differences between the two concert-viewing configurations were accompanied by differences in social presence, and that social presence statistically accounted for part of the differences in audience responses, the study provides preliminary evidence that social presence may be one psychological process associated with immersive concert experience. It also supports the view that virtual concerts should be understood not only as digital performances, but also as mediated social events. Importantly, the present study does not establish that this pattern is specific to concert viewing. Immersive presentation may increase social presence or emotional engagement across many forms of mediated content. Because the study did not include a non-musical or non-concert control stimulus presented under comparable immersive and non-immersive conditions, the extent to which the observed pattern reflects a concert-specific psychological process remains an open empirical question.

From an applied perspective, the present findings suggest that social presence deserves consideration in the design of virtual concert experiences. However, because the present experiment did not independently manipulate specific social or technological features, it cannot determine which design elements are most effective for increasing social presence. Future factorial experiments could separately manipulate features such as visible audience cues, avatar-based co-presence, performer–audience interaction, viewpoint control, and display modality. Such features may help transform virtual concerts from one-way broadcasts into socially meaningful music experiences. This is particularly relevant for virtual idols, online fan communities, and livestreamed music events, where audience engagement often depends on mediated interaction and community visibility.

Several limitations qualify the interpretation of the findings. First, the experimental contrast bundled multiple technological features. The immersive condition combined a head-mounted display, 360-degree presentation, a wider visual field, and head-contingent viewpoint exploration, whereas the standard condition used a computer screen, two-dimensional video, and a fixed viewpoint. The observed differences therefore represent the effect of the overall viewing configuration and cannot be attributed independently to any single component. In addition, because the study did not include a comparable non-concert immersive-media condition, it cannot establish that the observed pattern is specific to concert contexts.

Second, the study relied on a single 4-min performance excerpt. The findings therefore concern immediate responses to a brief viewing episode rather than a complete concert experience. Full-length concerts may involve dynamic processes absent from the present stimulus, including transitions between songs, performer–audience interaction, accumulating anticipation, fatigue, and changing emotional intensity. Because only one performer and performance segment were examined, generalization to other performers, musical genres, songs, and production formats also requires caution.

Third, social presence and the audience outcomes were assessed during the same post-exposure measurement session. Although viewing condition was randomly assigned, social presence itself was not experimentally manipulated. The significant indirect effects therefore provide evidence of statistical mediation but do not establish social presence as a causal mediator. Future studies could directly manipulate social-presence cues while holding display characteristics constant or use temporally separated measures to strengthen causal inference.

Fourth, although the study assessed several pre-exposure characteristics, including familiarity with the performer and song, pre-existing fan status, and previous exposure to the stimulus, emergent fan identification was measured only after stimulus exposure. The study therefore cannot estimate within-person change from a pre-exposure level of performer-specific fan identification. Accordingly, this outcome should be interpreted as an immediate, state-like post-exposure identification response rather than as evidence that stable fan identity was newly formed during the experiment. Future studies should include matched pre- and post-exposure measures of performer-specific fan identification and longitudinal follow-up to determine whether immediate identification responses persist or develop into more stable forms of fandom.

In conclusion, participants exposed to the immersive viewing configuration reported stronger social presence, enjoyment, emotional arousal, and post-exposure fan-related identification than those who viewed the same performance in the standard two-dimensional configuration. Social presence statistically accounted for part of these between-condition differences. These findings identify social presence as a promising psychological correlate of immersive concert experience, while further research is needed to isolate the contributions of specific technological features and to establish whether social presence plays a causal and concert-specific mediating role.

## Data Availability

The raw data supporting the conclusions of this article will be made available by the authors, without undue reservation.
